# A Personalized Self-Management Rehabilitation System for Stroke Survivors: A Quantitative Gait Analysis Using a Smart Insole

**DOI:** 10.2196/rehab.5449

**Published:** 2016-11-08

**Authors:** Richard John Davies, Jack Parker, Paul McCullagh, Huiru Zheng, Chris Nugent, Norman David Black, Susan Mawson

**Affiliations:** ^1^Computer Science Research InstituteFaculty of Computing and EngineeringUlster UniversityBelfastUnited Kingdom; ^2^School of Health and Related ResearchUniversity of SheffieldSheffieldUnited Kingdom

**Keywords:** ambulatory monitoring, gait, rehabilitation, self-management, smart insole, stroke

## Abstract

**Background:**

In the United Kingdom, stroke is the single largest cause of adult disability and results in a cost to the economy of £8.9 billion per annum. Service needs are currently not being met; therefore, initiatives that focus on patient-centered care that promote long-term self-management for chronic conditions should be at the forefront of service redesign. The use of innovative technologies and the ability to apply these effectively to promote behavior change are paramount in meeting the current challenges.

**Objective:**

Our objective was to gain a deeper insight into the impact of innovative technologies in support of home-based, self-managed rehabilitation for stroke survivors. An intervention of daily walks can assist with improving lower limb motor function, and this can be measured by using technology. This paper focuses on assessing the usage of self-management technologies on poststroke survivors while undergoing rehabilitation at home.

**Methods:**

A realist evaluation of a personalized self-management rehabilitation system was undertaken in the homes of stroke survivors (N=5) over a period of approximately two months. Context, mechanisms, and outcomes were developed and explored using theories relating to motor recovery. Participants were encouraged to self-manage their daily walking activity; this was achieved through goal setting and motivational feedback. Gait data were collected and analyzed to produce metrics such as speed, heel strikes, and symmetry. This was achieved using a “smart insole” to facilitate measurement of walking activities in a free-living, nonrestrictive environment.

**Results:**

Initial findings indicated that 4 out of 5 participants performed better during the second half of the evaluation. Performance increase was evident through improved heel strikes on participants’ affected limb. Additionally, increase in performance in relation to speed was also evident for all 5 participants. A common strategy emerged across all but one participant as symmetry performance was sacrificed in favor of improved heel strikes. This paper evaluates compliance and intensity of use.

**Conclusion:**

Our findings suggested that 4 out of the 5 participants improved their ability to heel strike on their affected limb. All participants showed improvements in their speed of gait measured in steps per minute with an average increase of 9.8% during the rehabilitation program. Performance in relation to symmetry showed an 8.5% average decline across participants, although 1 participant improved by 4%. Context, mechanism, and outcomes indicated that dual motor learning and compensatory strategies were deployed by the participants.

## Introduction

The global incidence of stroke is set to escalate from 15.3 million to 23 million by 2030 [1]. In the United Kingdom, stroke is the largest cause of disability [2] resulting in a cost to the economy of £8.9 billion a year [3]. It is estimated that following a stroke, only 15% of people will gain complete recovery for both the upper and lower extremities [4]. Walking and mobility are prominent challenges for many survivors who report the importance of mobility therapy [5]. Nevertheless, rehabilitative service needs cannot always be met and therefore initiatives that focus on patient-centered care promoting long-term self-management remain at the forefront of service redesign [6].

The adoption of technological solutions allows for patient and carer empowerment and a paradigm shift in control and decision-making to one of a shared responsibility. It also has the potential to reduce the burden for care professionals, and support the development of new interventions [7]. Incorporating technology into the daily lives of stroke survivors can be achieved by maintaining high levels of usability, acceptance, engagement, and removing any associated stigma involved with the use of assistive technology [8].

Technological aids for poststroke motor recovery hitherto have required the use of expensive, complex, and cumbersome apparatus that have typically necessitated the therapist to be present during use [9,10]. Recently, inexpensive, wearable, commercially-available sensors have become a more viable option for independent home-based poststroke rehabilitation [11,12]. A systematic review by Powell et al [13] identified a number of wearable lower-limb devices that have been trialed, such as robotics [14-16], virtual reality [16], functional electrical stimulation (FES) [17,18], electromyographic biofeedback (EMG-BFB) [19,20], and transcutaneous electrical nerve stimulation [21]. Of the identified trials exploring improvements in the International Classification of Functioning (ICF) domain of activities and participation, only 1 [21] found significant improvements. Studies that adopt a positivist randomized controlled trial paradigm often fail to give sufficient consideration as to how intervention components interact [22]. Indeed, creating and developing technological solutions for complex long-term conditions is challenging and requires multiple stakeholder input [23].

The Self-management supported by Assistive, Rehabilitation and Telecare Technologies consortium explored rehabilitation for stroke survivors focusing initially on the use of wearable sensors to support upper limb feedback on the achievement of functional goals [24-30]. User interface design, the practicalities surrounding deployment, and the ability of the participants to interact with the technology were explored [24].

The intervention model for the stroke system was based around a rehabilitation paradigm underpinned by theories of motor relearning and neuroplastic adaptation, motivational feedback, self-efficacy, and knowledge transfer [31-34]. In order to enhance and strengthen previous research, a realist evaluation [35] was adopted to evaluate the final personalized self-management rehabilitation system (PSMrS) prototype in order to gain an insight into the value, usability, and potential impact on an individual’s ability to self-manage their rehabilitation following a stroke [36].

The aim of this work was to understand the conditions under which technology-based rehabilitation would have an impact (outcome) on the motor behavior of the user—more specifically what would work for whom, in what context, and in what respect utilizing a realist evaluation framework [35]. This paper addresses this by focusing on the impact smart insole technology has on participants at home. The impacts are assessed by analyzing a participants’ gait over time, which are then presented and discussed.

Futhermore, the rehabilitation system, its architecture, and technical components are presented along with the evaluation of the prototype with regards to the performance and usability of the system in the homes of stroke survivors.

## Methods

### Summary

The methodology was divided into 2 phases: the first was to design and develop a PSMrS for stroke survivors, and the second was to conduct a realist evaluation of the PSMrS involving stroke survivors (N=5) at home. Phase I was responsible for the design and development of a set of user requirements and to evolve the design through 3 development cycles. The realist evaluation took place in Phase II and quantitative results were obtained while the participants used the system at home. Table 1 provides an overview of participants’ details; the mean age of participants was 57 years (range 42-73 years). Participants self-reported their computer experience as either none (+), fair (++), or a lot (+++). All of the participants routinely used a functional electrical stimulation (FES) device to enhance or stimulate dorsiflexion on their weaker side. While using this insole, none of the users used their FES at the same time. The FES and smart insole could not be used together simultaneously due to the added difficulty of donning and doffing the 2 devices on the lower limb. In addition, there was potential for interference of 1 system with the other.

**Table 1:** Demographics of participants with stroke.

Participant ID	Age of participants with stroke/carer	Affected side	Time since stroke (months)	Computer experience	Walking aid (FES^a^)
1	63/57	R hemi	13	++	None
2	73/73	L hemi	18	+	Frame or tripod
3	45/44	R hemi	18	+++	None
4	60/60	L hemi	15	++	None
5	42/44	R hemi	12	++	None

^a^FES: functional electrical stimulation.

### Realist Evaluation

The realist evaluation [35] concerned aspects of the system that would facilitate behavior change associated with the self-management of rehabilitation. The evaluation systematically tested the context mechanism outcome configurations [37] by deploying the system in the homes of stroke survivors for a period of up to 7 weeks (Table 2).

#### Intervention

Participants (N=5) received training on how to use the system and had access to an electronic manual that contained instructional videos. Technical support was available via mobile phone from 9 am until 5 pm during weekdays. Each participant was asked to use the system as frequently and for as long as they desired for the duration of the intervention (N=5, mean=41 days, range 27-50). This allowed researchers to evaluate the variation in desired frequency and intensity of use. All of the participants received feedback following each walking activity. The interventions included both upper and lower limb exercises to promote a more comprehensive and holistic approach to the rehabilitation process.

**Table 2:** Two quantitative context mechanisms outcome configurations referred to as translating feedback and individual feedback for the personalized self-management rehabilitation system (PSMrS).

Feedback	Context	Mechanism	Outcome
Translating feedback	A system that translates biomechanical data through feedback.	The use of the PSMrS will facilitate the translation of biomechanical data which might enable the user to interpret their symptoms.	An understanding of symptoms and change in symptoms throughout the usage of the system. Measure: Qualitative data and quantitative Web-based data sources from insole.
Individual feedback	A system that provides individualized motivational feedback on the achievement of walking skill.	The use of the PSMrS might encourage increased intensity of practice with consequential neuroplastic changes.	Increased functioning and achievement of improved walking skill. Measure: Web-based quantitative data sources from insole.

#### Technology Deployed

**Figure 1:**
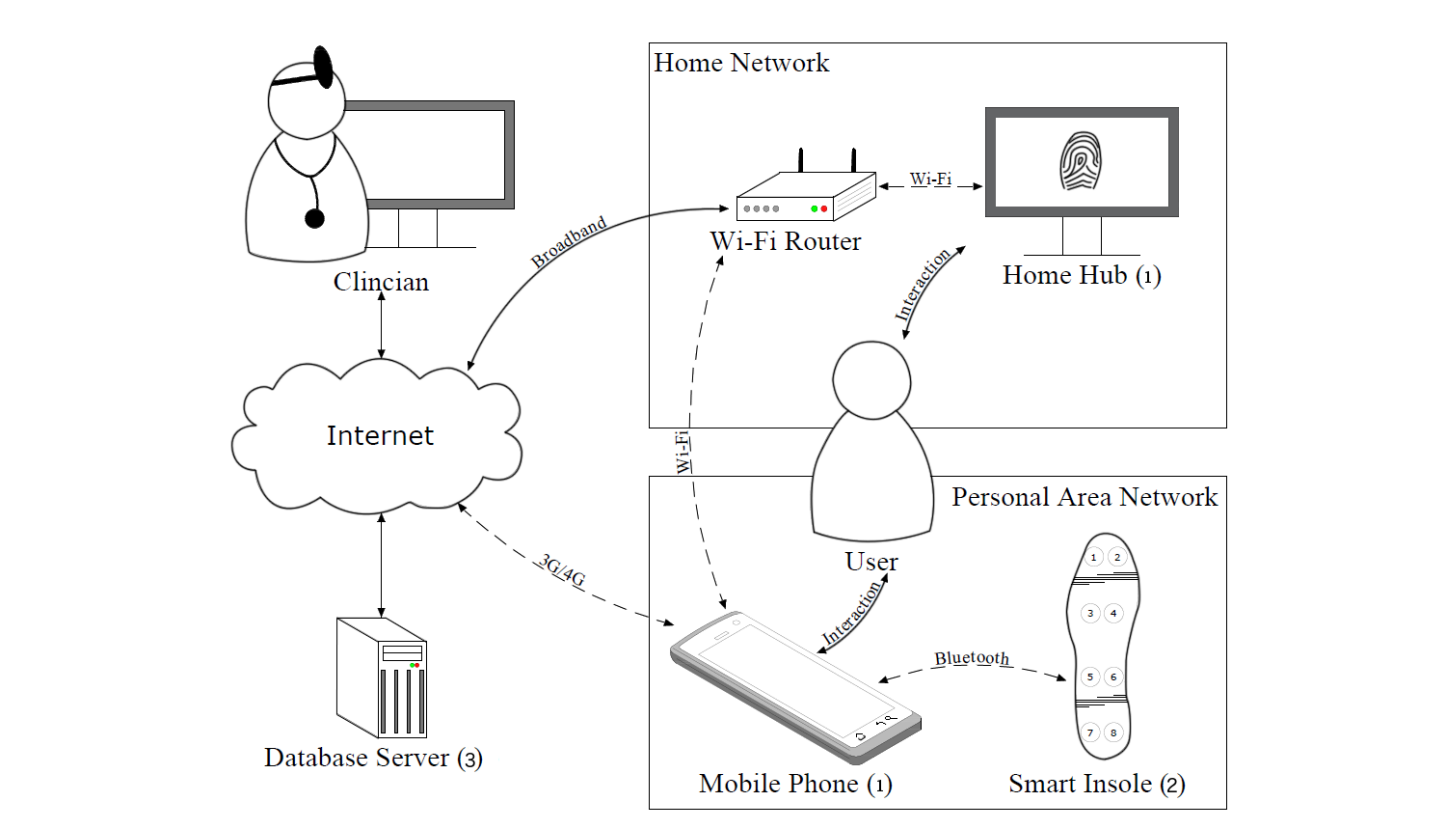
Technology infrastructure used to support the realist evaluation consisted of touch screen interactive components: (1) a smart insole produced by Tomorrow Options, (2) used to collect gait information, and (3) a server used to analyze data.

The technology used to support the realist evaluation is presented in Figure 1 and consists of 3 parts. First, the touch screen interactive computing components, which are a home hub and mobile phone. The home hub facilitated the presentation, collection, forwarding, and synchronization of data and information related to the rehabilitation process. The upper limb intervention was only available through the home hub while the lower limb intervention was available on both the home hub and mobile phone components. Second, the mobile phone was combined with the smart insole to form a personal area network to enable gait information to be collected in real time and subsequently stored on the mobile phone. The home hub enabled participants to visualize their walking data via feedback screens (Multimedia Appendix 1) and make any adjustments via self-management. Third, upload of data to the server facilitated researchers to further analyze beyond those performed in real time for the participants.

These interventions were directly mapped onto 2 primary features offered by the PSMrS. The first intervention involved the monitoring and feedback of a participant’s gait while performing walking activities. Walking activity was monitored by a smart insole that collected plantar foot pressure data, relating to a participant’s gait. The smart insole is a product called Walkinsense produced by Kinematix, Portugal (formerly Tomorrow Options, Sheffield, United Kingdom). Information such as number of heel strikes for both affected and nonaffected sides, symmetry, and speed were calculated, stored, and fed back to participants. The second intervention focused on providing participants with access to a library of both upper and lower limb exercises, for example reaching, sit-to-stand, and stepping. A personalized selection of library exercises was created for each participant. This selection of exercises was mapped on to a predefined list of goals that participants could choose from. Instructional videos were presented to participants to promote clarity on form and precision of movement as these are deemed to be important factors in rehabilitation.

The quality metrics chosen for feedback were the number of heel strikes and symmetry on the affected side. Feedback was provided through 2 screens, one for heel strikes and one for symmetry as presented in Multimedia Appendix 1.

Participants were given the opportunity to assess their personalized feedback and make appropriate changes where they deemed it necessary to do so, according to the principles of self-management.

#### Data Processing

**Figure 2:**
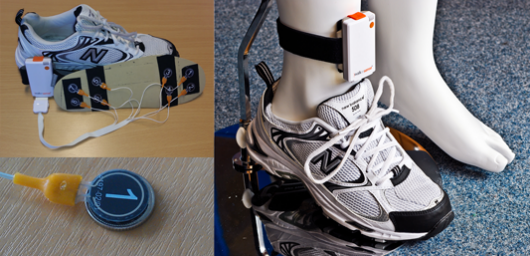
Walkinsense device. Top left: force sensitive resistors showing a typical layout configuration; bottom left: the size of a force sensitive resister in relation to a UK 5 pence piece; and right: attachment of devices to lower limb on a manikin.

The PSMrS uses a personal area network that comprises of a smart insole that transmits data in real time via a Bluetooth channel connected to a mobile phone for persistence. The smart insole, as presented in Figure 2, comprises a network of 8 force-sensitive resistors per foot or insole and samples data at a frequency of 100 Hz at a resolution of 8 bits. The data were captured in real time and uploaded to a server for further analysis for each walking activity.

The time series data were analyzed to extract high-level information such as the length of the walking activity, number of steps, speed, number of heel strikes, and symmetry information. Once calculated, all of the metrics are persisted to a database table to be accessed for feedback to the stroke participant. A subsequent analysis was carried out across all of the participants to assess any trends, patterns of use, and to identify any strategies adopted.

#### Feature Extraction Algorithm

**Figure 3:**
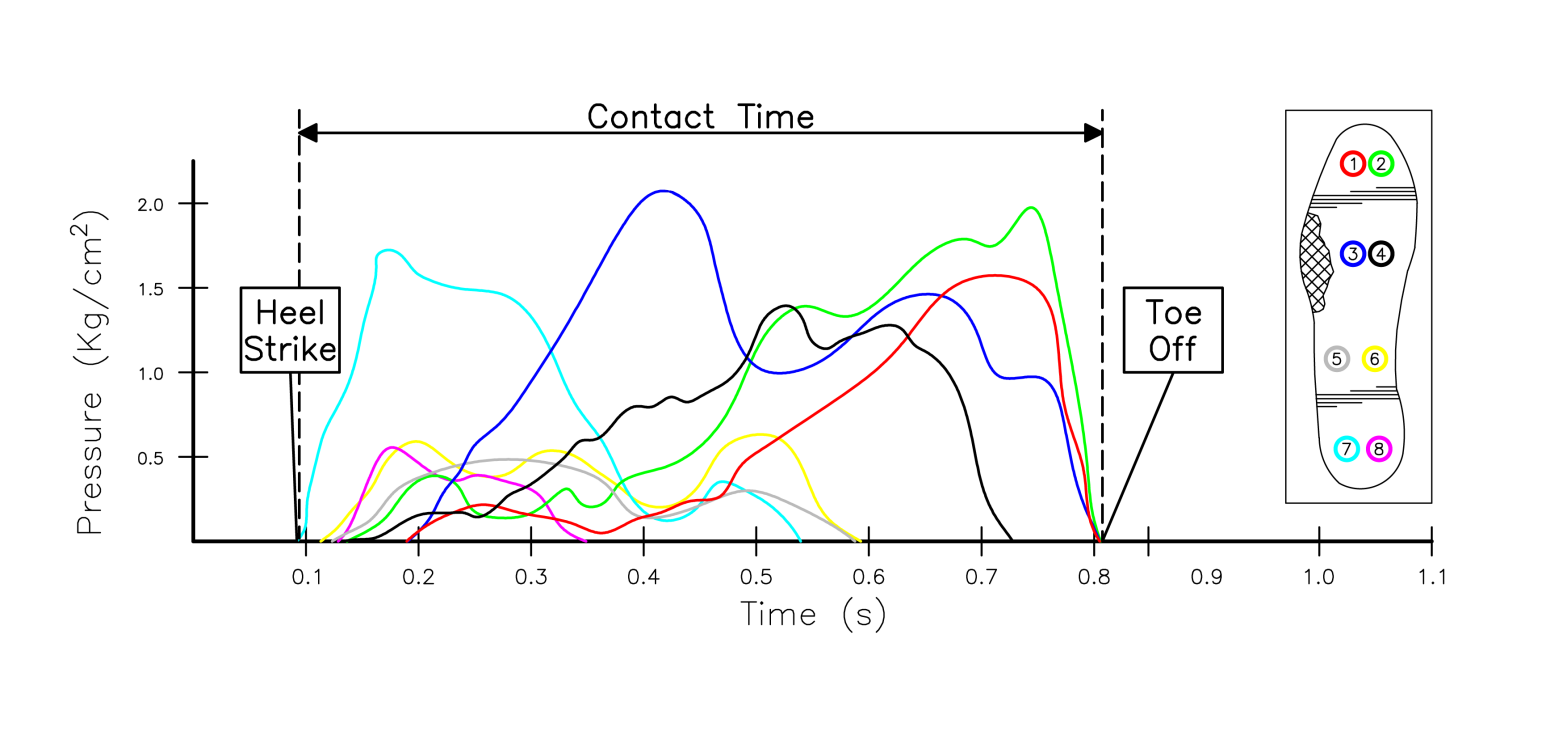
Time series data showing pressure distribution for a single foot strike.

Time series data from 8 sensors were plotted for each insole allowing the data to be manually inspected and annotated to verify results (Figure 3). In order to process high-level features such as number of steps and symmetry, the lower level features had to be derived first. These features identify fundamental gait events such as the point when the foot contacts and leaves the ground (Table 3).

The algorithm works by cycling through the time series data while detecting periods of pressure contact with the ground. These time periods are extracted to form a “step object” that is analyzed to produce the sublevel features listed in Table 3. The high-level features are calculated by analyzing all step objects produced for the whole walking activity. Over time, with significant reuse, sensors can potentially yield out of range values or become faulty. As part of the symmetry calculation, the algorithm takes into consideration any faulty sensors and removes them through a matching process with the opposite foot. This ensures that faulty sensors, should they occur, are not responsible for biasing or invalidating the symmetry calculation.

**Table 3:** Features and their description that were generated from the raw data collected from the insole.

Feature	Description	Units
Toe off	Time and sensor location when the foot leaves the ground	ms
Heel strike	Time and sensor location when the foot strikes the ground	ms
Contact time	Overall ground contact time of the foot	ms
Average pressure	Pressure exerted across the entire foot during contact time	kg/cm^2^

## Results

### Summary

The results focus on the analysis of the quantitative data collected during the realist evaluation. From this, we assess if there were any significant improvements in performance in relation to walking activity and what area these improvements might relate to. The data were split into 2 halves: if a participant performed 20 walking activities throughout the entire realist evaluation, then the first 10 of these would constitute the first half and therefore represent baseline data. Rehabilitation markers were identified in relation to a participant’s gait—these were number of heel strikes, symmetry, and speed. Heel strike information was split into 2 parts to accommodate participant’s affected versus nonaffected side.

The results across all 5 participants within the evaluation period demonstrated that on average, performance in relation to speed and heel strikes on a participant’s affected side improved by 9.8% and 8.8%, respectively. In contrast, performance in heel strikes and symmetry on participant’s nonaffected side decreased by 9.9% and 8.5%, respectively. Although these results were averaged across all the participants, this common pattern was evident (where participants’ favored heel strikes on their affected side and increased speed) for 4 out of the 5 participants.

Participants were given feedback on 2 metrics: symmetry, and heel strikes on their affected side. The goals for these 2 metrics were personalized to 100% for heel strikes on their affected side and to 50% for symmetry. Although the participants’ speed was not used as a feedback metric, information on this was collected. On average, across all 5 participants, speed of walking showed a marked increase of 9.8% during the evaluation period.

**Figure 4:**
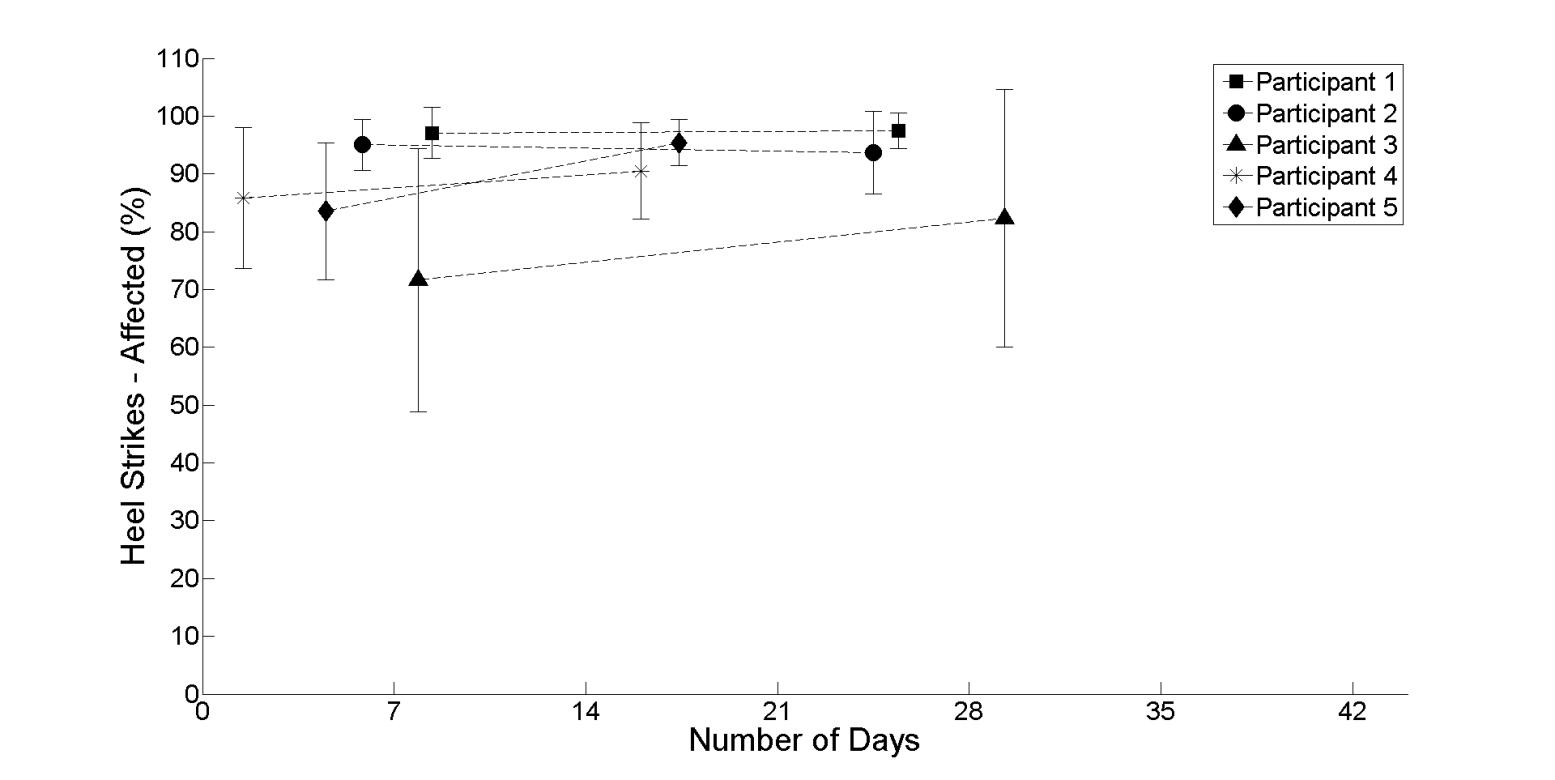
The average between the first and second half of the realist evaluation for heel strikes on the participants’ affected side starting at day 1.

**Figure 5:**
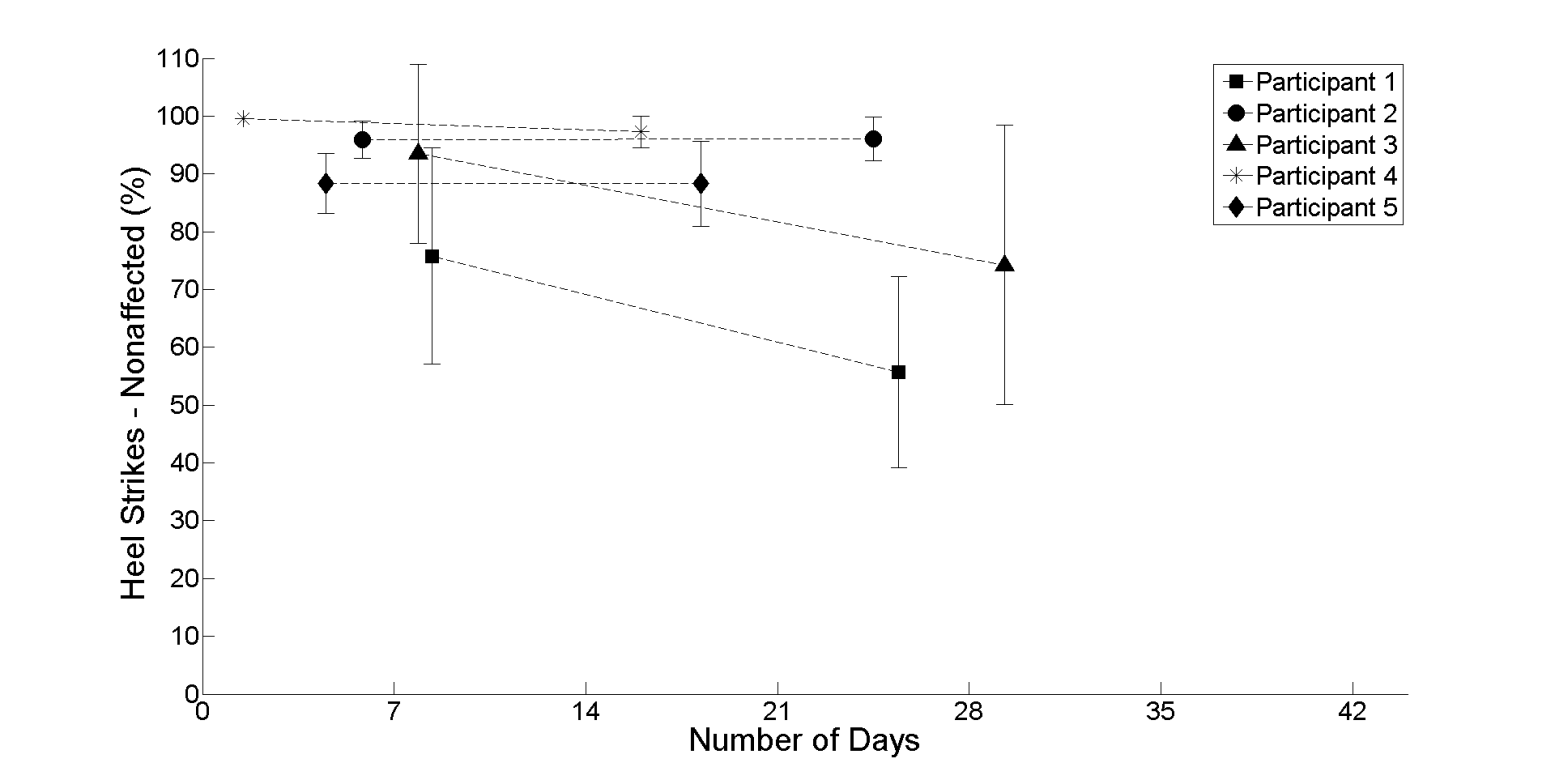
The average between the first and second half of the realist evaluation for heel strikes on the participants nonaffected side starting at day 1.

**Figure 6:**
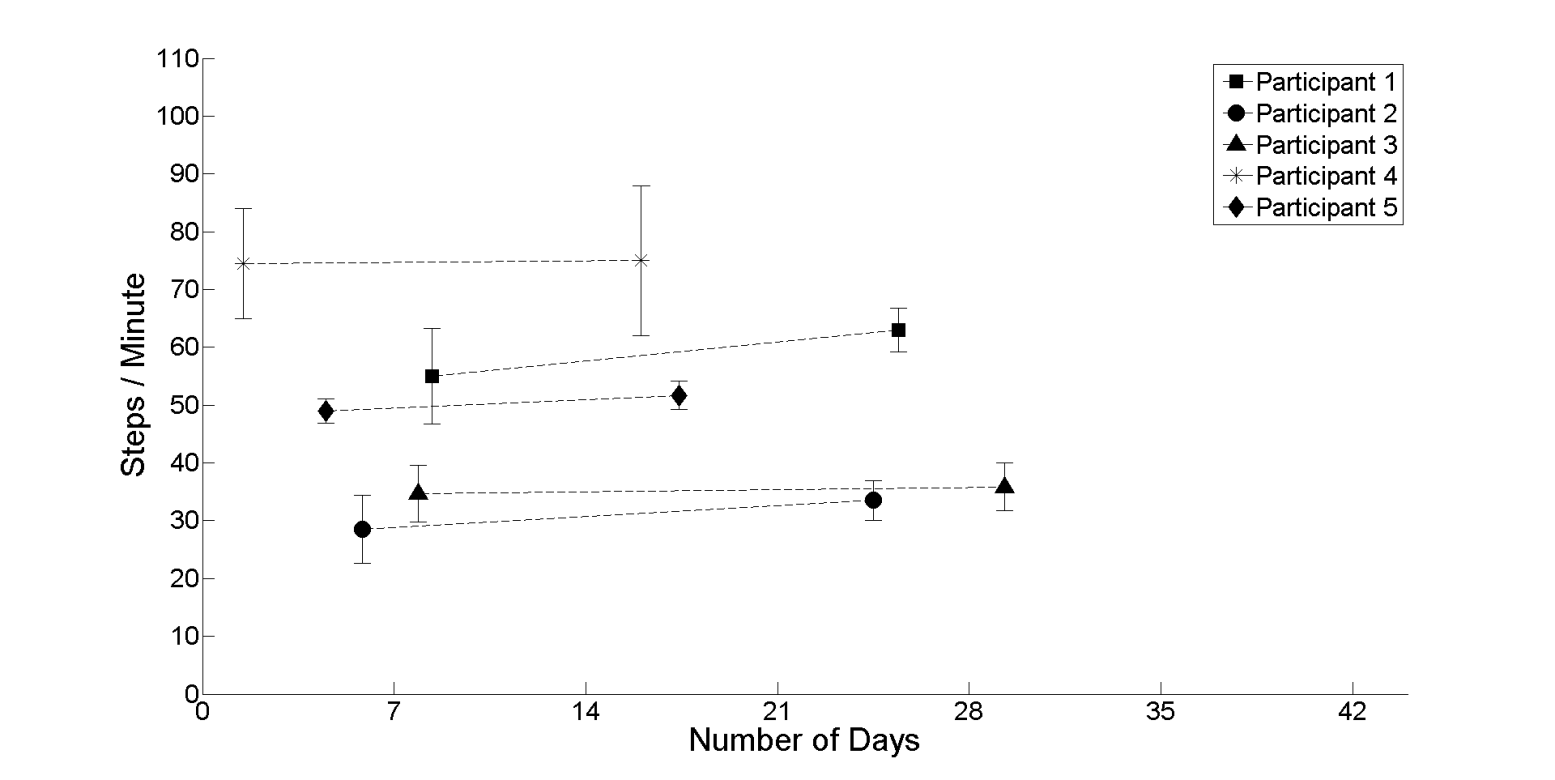
The average between the first and second half of the realist evaluation for steps/minute (speed) for all participants starting at day 1.

**Figure 7:**
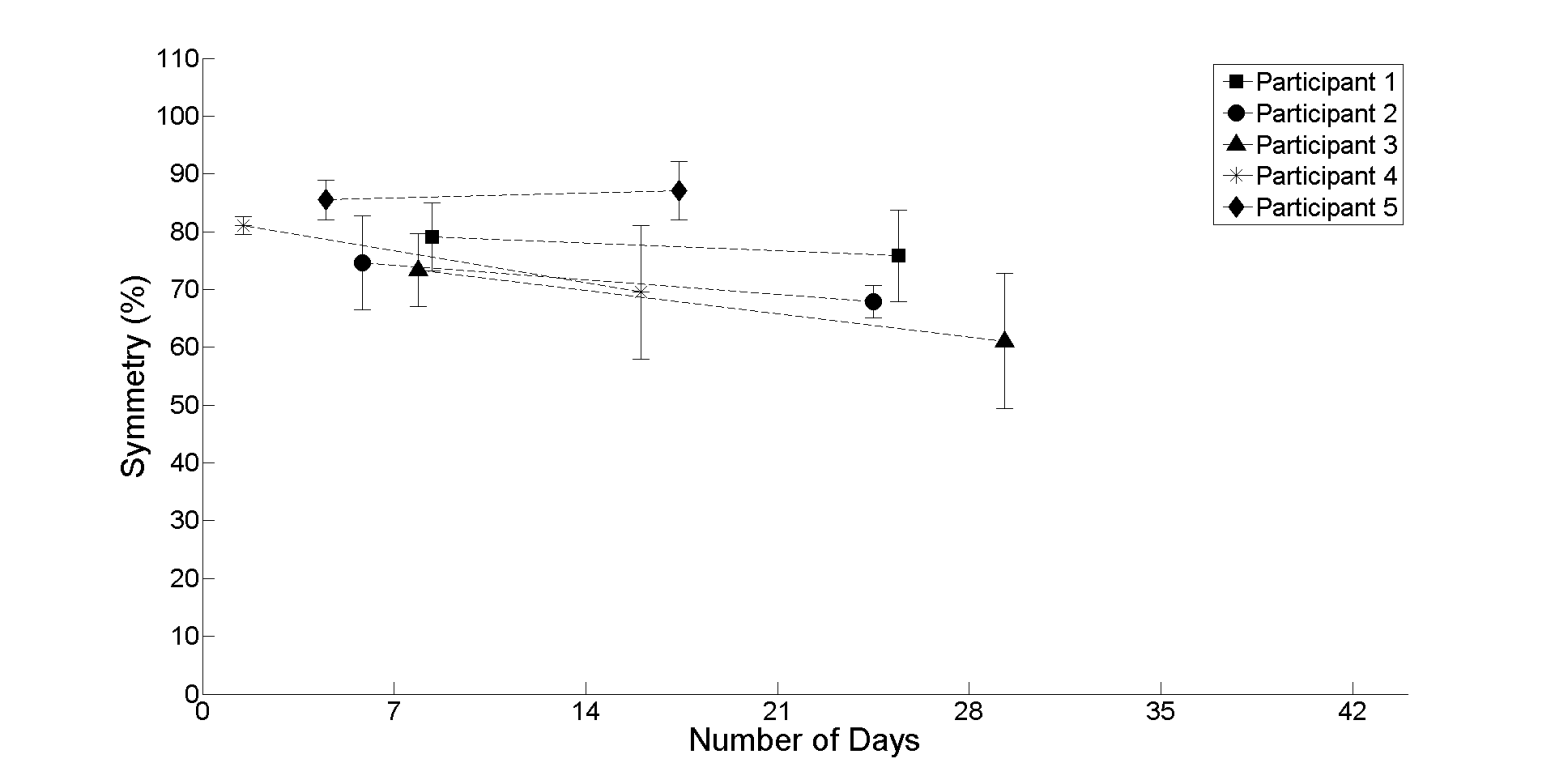
The average between the first and second half of the realist evaluation of symmetry for all participants starting at day 1.

Figures 4-7 provide further insight into each of the metrics showing the change between the first and second halves of the realist evaluation. The symbols (square, circle, triangle, asterisk, and diamond) represent the average at the midpoint of the first and second half of the realist evaluation. A pattern has emerged for each of the 4 metrics: a marginal upward or leveling tendency for heel strikes on participant’s affected side (Figure 4), a marginal decline for heel strikes on participants’ nonaffected side (Figure 5), an upward or leveling tendency for speed (Figure 6), and a consistent decline for balance (with the exception of participant 5; Figure 7).

**Figure 8:**
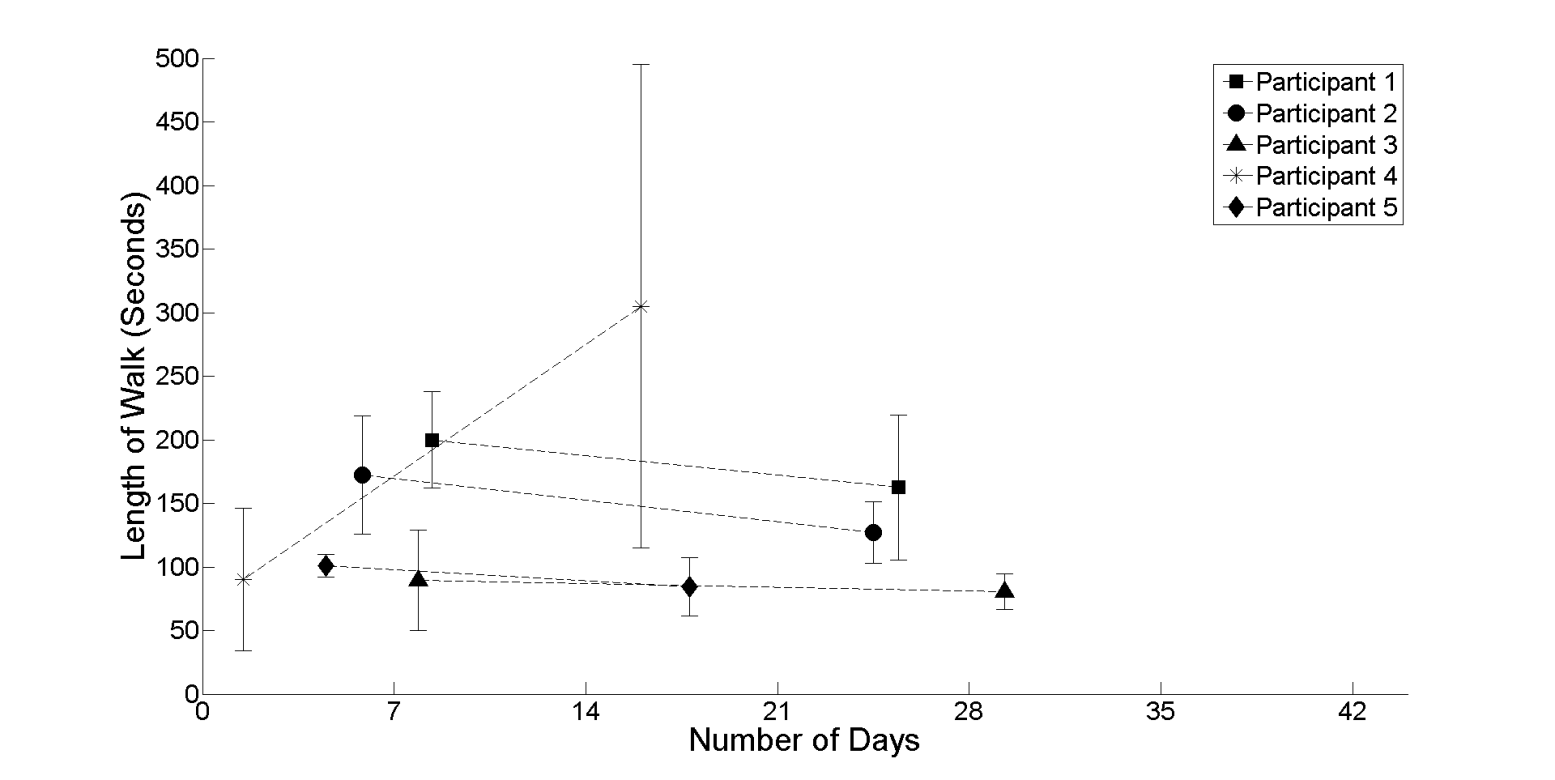
High level summary information in relation to the length of walk in seconds. With the exception of participant 4, it shows a very gradual decline in intensity of use.

**Figure 9:**
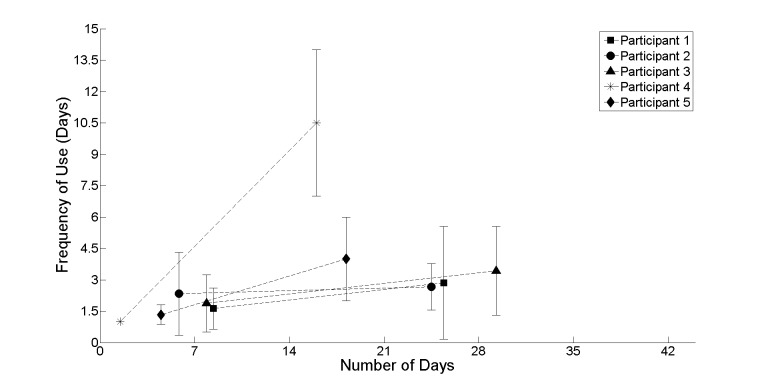
The frequency with which participants used the system irrespective of how intense that use was. This indicates an intention to perform a daily walk. It shows a decline in frequency of use from the first to second half of the realist evaluation.

In addition, the analysis focused on participants’ compliance, how often they used the system (Figure 9), and their intensity of use (length of walks; Figure 8). Together these metrics can be used to inform how participants were motivated throughout the realist evaluation and provide some indication in relation to participants’ stamina and ability to recover.

Looking at the group of participants as a whole, it is probable that the pattern of use by participant 4 can be treated as an outlier. A closer analysis of participant 4 indicates that frequency of use declined from once per day to over once every 10 days. Coupling this pattern of infrequent use with a marked increase in the intensity of use (length of walks) from 90 seconds to 305 seconds could be an anomaly within the cohort profile. The remainder of the cohort, participants 1, 2, 3, and 5, has a similar pattern of use indicating both a slight decline in frequency and intensity of use. The rationale or explanation behind this can be linked to an adoption for new technologies for which there are many underlying reasons [38]. In particular, the novelty factor and how this could wear off during the first few times of use. Taking a closer look at these patterns of use does support this explanation as the first few times of use provide the marked increase necessary to create the slight decline viewed across participants 1, 2, 3, and 5.

The results from this paper focus on the quantitative data collected during the realist evaluation. Furthermore, information and details of qualitative results are published by Mawson [36]. Participant 2 described how the individual feedback scores helped to see progress towards recovery: “It makes me feel like I’m making progress. I’m going down that road to full recovery.” When asked about achieving a lower score than a previous attempt, participant 4 suggested that this inspired them to try again: “It made me want to do it again to better it, yeah.”

## Discussion

### Principal Findings

Although the results presented in this paper are not considered to be conclusive across a wider population of stroke participants, we have been able to add to existing literature by embedding our methods within an innovative realist evaluation methodology and by exploring changes in walking patterns within the real-world context of home-based rehabilitation. Although we have intervened by removing the FES, the results obtained can be clearly attributed to the technology being evaluated.

Theoretically, increased intensity together with motivational feedback should result in motor learning and neuroplastic adaptations. Nevertheless, the development of compensatory strategies has been documented in both rehabilitation literature [39-41] and in research findings [10,42]. As Kirker suggests, compensatory patterns are adaptive movements that reflect the central nervous system lesion, the structure of the motor system, and the environmental demands placed on the individual.

It seems a common strategy was adopted by 4 out of 5 participants to improve heel strikes on their affected side at the detriment of heel strikes on their nonaffected side. To achieve this strategy, participants compensated their balance by placing more weight and control on their nonaffected limb. Only participant 5 was able to improve heel striking on their affected limb while also improving their balance. Essentially this is a compensation strategy [41] whereby the nonaffected limb is used to compensate for balance and proper heel striking function to perform better on the rehabilitation feedback scores. This dual motor learning, compensation strategy previously described by Kirker et al [10] can be addressed with further research through the development of a new context mechanism outcome. Interestingly, all 5 participants increased their speed and for participants 1 and 2, this was relatively a significant increase of 23.8% and 17.5% respectfully. This increase in speed is interesting for a number of reasons. Participants didn’t receive any feedback on how they were performing in relation to speed, so the increase in speed is not related to any feedback or encouragement they would have received. Secondly, it seems counterintuitive to increase your speed to perform better at heel striking and balance yet all 5 participants did so. Speed is a metric that requires more research into its contribution and effects on the gait of stroke survivors at home.

A number of common patterns or strategies adopted in this study have been identified. It is clear that all participants compensated by not performing well on their good side to perform better on their affected side (for heel strikes). The results indicate that this compensation was almost a direct trade-off with an 8.8% increase versus a 9.9% decrease, respectively. In addition to this compensation strategy it is evident that participants’ symmetry was also effected resulting in a proportionate decrease of 8.5%. This trade-off or dual strategy has been reported before [42] where it was shown that some stroke survivors improved functionally by using compensatory strategies, suggesting that factors predicting which participants use compensatory strategies needs further study. Whilst confirming and refining the original context mechanism outcomes, a further context mechanism outcome has therefore emerged from the evaluation linking the PSMrS directly to the dual strategy by increasing the demand on the individuals because of the increased intensity, goal planning, and the feedback screens (refer to Table 4).

Monitoring and providing feedback on key metrics related to improved quality of gait, aims to promote behavior change through goal setting, feedback, and self-management which map on to behavior change techniques [43]. In terms of behavior change, feedback scores had a significant effect as there was a focus toward achieving better results for heel strikes on their affected side versus their symmetry or heel strikes on their nonaffected side (Table 5). In addition, increasing speed may indicate a behavior change toward higher confidence levels which can be confirmed by the qualitative research carried out by Mawson [36]. Furthermore, research should be conducted to confirm these assumptions as speed was not used as feedback.

The results indicate that the pattern of use in terms of frequency and intensity of use declined slightly from the first and second half of the realist evaluation. Future work would incorporate a mechanism to manage and maximize participant motivation by aligning mood and wellbeing feedback into overall feedback scores to avoid situations where participants become deflated. In addition, gamification elements could be added to provide enhanced motivation; these could take the form of levels or badges to accomplish milestones.

**Table 4:** Modified (translating feedback and individual feedback) and newly emerging context mechanism outcome (dual motor learning).

Description	Context	Mechanism	Outcome
Translating feedback	(Modified) A system that translates accurate, reliable quantitative biomechanical data through feedback.	The use of the PSMrS will facilitate the translation of biomechanical data which might enable the user to interpret their symptoms.	An understanding of symptoms and change in symptoms throughout the usage of the system. Measure: Qualitative data and quantitative Web-based data sources from insole.
Individual feedback	(Modified) A system that provides accurate, reliable quantitative individualized motivational feedback on the achievement of walking skill.	The use of the PSMrS might encourage increased intensity of practice with consequential neuroplastic changes.	Increased functioning and achievement of improved walking skill. Measure: Web-based quantitative data sources from insole.
Dual motor learning	(New) A system that increases environmental demands on the individual.	(New) The use of the PSMrS might encourage functional recovery achieved through dual motor learning and compensatory strategies.	(New) Increased walking skill with an increase in compensatory strategies. Dual strategy adopted. Measure: Web-based quantitative data sources from insole.

**Table:** Performance for all 5 participants indicates relative and contrasting scores for heel strikes on both sides, balance, and speed. The relative scores are obtained by contrasting the first and second half usage during the realist evaluation.

Participant ID	Heel strikes (Affected)	Heel strikes (Nonaffected)	Balance (Affected)	Speed
1	+0.7%	−32.3%	−7.4%	+23.8%
2	−1.4%	+0.1%	−9.1%	+17.5%
3	+29.0%	−11.5%	−15.8%	+1.5%
4	+5.4%	−2.3%	−14.2%	+0.7%
5	+10.5%	−3.7%	+4.0%	+5.5%

### Limitations

The first limitation is the lack of a nonintervention baseline data to compare and contrast against the realist evaluation. The study is therefore limited to comparing and contrasting data within the first and second halves of the realist evaluation. The second limitation relates to both the number of participants and duration of the study which could be extended to establish significance to the results. Future work aims to address this by evaluating this approach and technology through a randomized controlled trial.

### Conclusions

This research aimed to gain a deeper insight into the impact of innovative technologies under the context of home-based rehabilitation for stroke survivors. In this study, the authors present the results from a realist evaluation that focuses on the introduction of smart insole technology to a cohort of (N=5) stroke participants. The study focuses on the quantitative data obtained and analyzed from walking activity data generated over a 2-month period in participants’ homes using realist evaluation methodology. The results have provided further insight into how stroke participants perform during walking activities at home without direct instruction and supervision. The results show that participants may be willing to compensate and sacrifice performance in symmetry or balance in favor of heel strikes on their affected side. Speed was also identified as a metric that exhibited a marked increase through higher confident levels after using the smart insole technology for a short period of time which was an unexpected result. Motivational aspects of the system should also be improved to encourage higher levels of frequency and intensity of use.

## Multimedia Appendix 1

Symmetry or balance feedback screen (top), Heel strikes feedback screen (bottom).
