# Supplementary figures and images for: A Personalized Self-Management Rehabilitation System for Stroke Survivors: A Quantitative Gait Analysis Using a Smart Insole
Source: JMIR Rehabil Assist Technol. 2016 Nov 8;3(2):e11. doi: 10.2196/rehab.5449 (PMC5454559; doi:10.2196/rehab.5449)

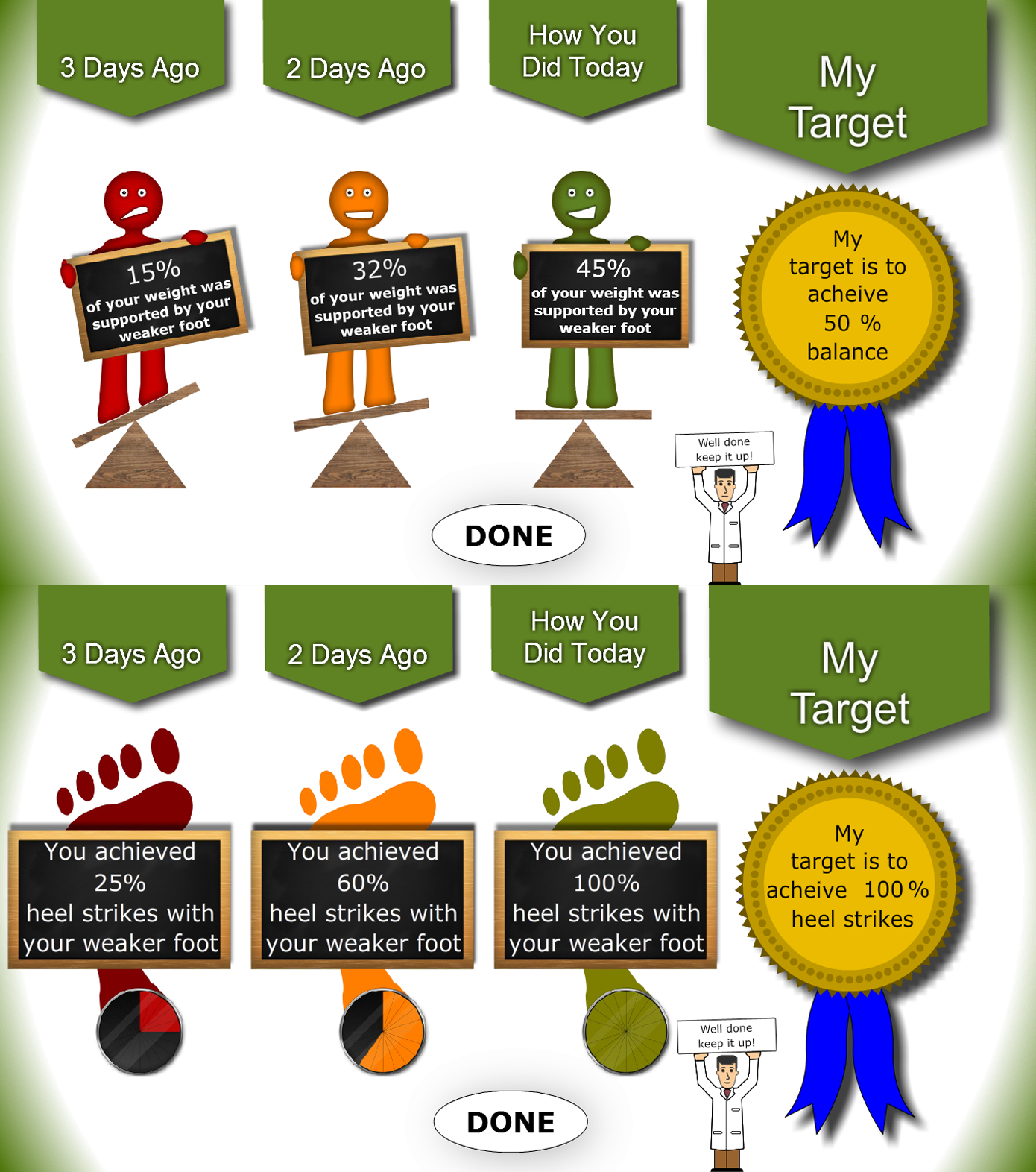

Supplement: Supplementary file 1 [file rehab_v3i2e11_app1.png]
